# Tannin tolerance lactic acid bacteria screening and their effects on fermentation quality of stylo and soybean silages

**DOI:** 10.3389/fmicb.2022.991387

**Published:** 2022-09-15

**Authors:** Lin Gao, Xiang Guo, Shuo Wu, Dandan Chen, Liangfa Ge, Wei Zhou, Qing Zhang, Ruiqi Pian

**Affiliations:** College of Forestry and Landscape Architecture, Guangdong Key Laboratory for Innovative Development and Utilization of Forest Plant Germplasm, State Key Laboratory for Conservation and Utilization of Subtropical Agro-Bioresources, Guangdong Province Research Center of Woody Forage Engineering Technology, Guangdong Research and Development Centre of Modern Agriculture (Woody Forage) Industrial Technology, South China Agricultural University, Guangzhou, China

**Keywords:** lactic acid bacteria, tannic acid, tannin tolerance, fermentation quality, *in vitro* rumen fermentation

## Abstract

Some excellent legume forages are difficult to ensile naturally due to their high buffering capacity and low water-soluble carbohydrate content. This may cause serious problems like proteolysis. In the present study, strains of lactic acid bacteria with high acid productivity and high tannin tolerance were screened from different silages and combined with tannic acid (TA) as an addition to ensiling. The screened strains were identified as *Lactobacillus plantarum* (LP), with four of these strains then selected for their high tannin tolerance. *Stylosanthes guianensis* and whole-plant soybean (WPS) were ensiled with 1 and 2% (fresh matter basis) TA, four LP strains alone (6 log_10_ colony forming units per gram of fresh matter), or TA combined with LP strains. Fermentation parameters and *in vitro* rumen fermentation characteristics were analyzed after 30 days of fermentation. The results showed that TA + LP can be used to reduce pH values (*P* < 0.01), non-protein nitrogen (*P* < 0.01), and ammonia-nitrogen (*P* < 0.01). The *in vitro* crude protein digestibility of WPS silage was also decreased with the addition of TA + LP (*P* < 0.01). These results indicate that the addition of TA combined with tannin tolerance LP strains may improve the fermentation quality of legume silage, especially for reducing proteolysis.

## Introduction

With the increasing development of the livestock industry and consumer demand for meat and dairy products, the demand for feed has increased gradually. Silage, an important feed for ruminants, needs to be constantly optimized using relevant technologies in order to improve the quality of fermentation. Ensiling is a wet storage fermentation method used to achieve long-term storage of fresh forage materials under anaerobic conditions (Nagle et al., 2020). This method has the advantages of simple operation and low cost and can also solve the problem of seasonal feed shortages. However, proteolysis inevitably occurs during the fermentation process, resulting in true protein (TP) being broken down and converted into non-protein nitrogen (NPN) mainly ammonia-nitrogen (NH_3_-N), thereby decreasing nutrient content (Wang et al., 2021). Some excellent legume forages, such as *Stylosanthes guianensis* (stylo) and *Glycine max* (soybean) are used as protein supplements in ruminants because of their high protein content. However, those plants always have a low water-soluble carbohydrate (WSC) content, high buffering capacity, or low counts of epiphytic lactic acid bacteria (LAB), which can lead to proteolysis (He et al., 2021). Silage additives are used most commonly to improve silage quality.

Lactic acid bacteria are often added to forage to improve silage fermentation, especially *Lactobacillus plantarum* (LP). These bacteria are able to metabolize WSC to lactic acid (LA) and rapidly decrease the pH value. They also produce silage with a lower nitrogen content and better dry matter (DM) recovery (Muck et al., 2018). Experiments have demonstrated that the NH_3_-N content of LP-added silage could be reduced from 6.4 to 4.1% TN (Du et al., 2021).

In recent years, tannic acid (TA) has also been tried as an additive to silage as it can inhibit the growth of harmful bacteria and also has the ability to reduce proteolysis (Herremans et al., 2019). There is evidence that TA combines with macromolecules such as proteins, structural carbohydrates, and starch during ensiling and rumen fermentation (Oliveira et al., 2009). Previous experimental studies have suggested that the NPN content, CH_4_ production, ratio of acetate to propionate, and NH_3_-N of the TA-added silage was reduced (Yang et al., 2017; He et al., 2020b). However, TA has a wide range of antibacterial activity and therefore it may inhibit the growth of LAB (Dong et al., 2019; He et al., 2020a).

While there may be benefits from using a single addition during ensiling, the addition of mixtures of different additives may have superior effects to a single addition. The addition of a mixture of TA with LAB strains with tannin tolerance to silage may reduce proteolysis of legume silage during ensiling. The aim of the present study was to screen LAB strains with tannins tolerance and determine the effects of treatments of stylo and whole-plant soybean (WPS) with TA and LAB either alone or in combination on fermentation characteristics. The study provides a new concept for silage additives, which involve adding a TA combination with LAB to further improve the fermentation quality and digestive characteristics of silage.

## Materials and methods

### Screening for tannin tolerance lactic acid bacteria

Four better LAB strains were identified by Zhang et al. (2016). The specific methods used in the present study were as follows. Samples of 20 g taken from either grape pomace, banana leaves, *Neolamarckia cadamba* leaves, or mangosteen fruit pericarps silages were mixed thoroughly with 180 mL of sterile saline and diluted to 10^–7^, based on 10-fold gradient dilutions. A 1 mL aliquot of the bacterium solutions from different gradients were transferred to 15 mL of solid Man, Rogosa and Sharpe (MRS) medium and cultured for 2 days at 30°C. Individual colonies were then picked and purified by repeated smearing on MRS medium. Individual colonies of dedicated strains were inoculated into MRS broth medium and incubated at 30°C for 2 days to obtain purified LAB solutions. About 100 μL aliquots of the purified bacterium solutions were transferred to 5 mL MRS liquid medium and cultured for 24 h. The pH values were then measured to evaluate acid production by the LAB. A total of 29 strains with high acid production were screened and then sequenced by the Guangzhou Gene denovo Biotechnology Co (Guangzhou, China). The sequences were aligned using basic local alignment search tool (BLAST).

Tannin tolerance tests were then carried out at different concentrations (0, 1, 2, and 4%) of TA (CAS:1401-55-4, purity ≥97.5%; Shanghai-Macklin, China mainland). A 100 μL aliquot of the identified bacterial liquid was added to 5 mL of sterilized MRS broth medium and incubated for 2 days at 30°C. The bacterial solution was diluted, and five gradients were taken (10^–7^–10^–11^ cfu/mL). A 100 μL aliquot of the diluted bacterial liquid was applied to MRS solid medium and incubated for 24 h at 30°C for single colony counting. The above procedures were repeated to obtain bacterial liquid that was then incubated for 2 days at 30°C, followed by centrifugation for 10 min at 4,000 rpm and washing once with saline. The liquid was centrifuged again and the supernatant gently decanted. The sediment was added to 5 mL of sterilized saline, mixed with a vortex, and diluted with saline to prepare solutions (10^–7^–10^–11^ cfu/mL). About 100 μL of these suspensions were then added to 5 mL of culture medium containing tannins (1 mL 20.0 g/L sterilized glucose solution + 4 mL sterilized saline with different concentrations, filtered using a 0.22 μm filter membrane). The cultures were then incubated at 30°C for 24 h, diluted and smeared on MRS solid medium. Colony counts were carried out after 2 days of incubation at 30°C. Finally, four LP strains with better tannin tolerance were selected and named LP1, LP2, LP3, and LP4. Of these, LP4 selected from mangosteen fruit pericarps silage had the highest viable count in the 4% tannin tolerance test.

### Raw materials and silage preparation

The stylo and soybean were planted in an experimental field at the South China Agricultural University (23°19′ N, 113°34′ E), Guangzhou, Guangdong Province, China, without the use of herbicides or fertilizer. WPS was collected at the drum stage, while stylo was collected by hand at the blooming stage. The plant materials were cut using a manual cutter (Model 9ZP-3.6, Kaiyue Machinery Company, China) into short sections of 2–3 cm length. After homogenization, the materials were divided into 16 groups. One of the groups was selected randomly and then divided into triplicates for determination of chemical composition and microbial populations of the raw material. The remaining 15 groups were treated for silage fermentation.

All the silage treatments were as follows: (1) with 1% (fresh matter basis) TA added; (2) with 2% TA added; (3) with LP added (6 log_10_ cfu/g fresh matter); (4) with LP and 1% TA added; (5) with LP and 2% TA added; and (6) without addition. Four selected LP strains (LP1, LP2, LP3, and LP4) were applied as silage additives.

The cut plant material and additives were mixed thoroughly to obtain approximately 100 g of silage material, packed into polyethylene bags (20 cm × 30 cm), and then sealed with a vacuum sealer (Lvye DZ280, Yijian Packaging Machinery Co., China). A total of 90 bags (2 species in 15 treatments with 3 replicates) were stored at room temperature (25–30°C) for 30 days. After 30 days of fermentation, the fermentation parameters and protein fractions of each treatment group were analyzed. In addition, because of its higher tannin tolerance, the treatment groups associated with LP4 were selected to measure *in vitro* gas production, dry matter digestibility, and crude protein digestibility.

### Analyses of microbial population and organic acids

After the specified fermentation time, the silage bags were opened, followed by even mixing of the materials in the bag. Twenty gram samples were then removed randomly from each bag, added to sterile 0.9% saline and then shaken. After about 15 min the supernatants were collected and serial diluted from 10^–1^ to 10^–6^ on a clean bench. The LAB and coliform bacteria (CB) were then cultivated and their number measured after incubation on de Man–Rogosa–Sharpe agar and violet red bile agar at 30°C for 2 days. Another 20 g of the samples were removed from the silage bags and mixed with 180 mL of distilled water and then kept at 4°C for 18 h before filtering through four layers of cheesecloth and filter paper. The pH of the filtrate was then determined immediately using a glass-electrode pH meter. The organic acids [LA, acetic acid (AA), propionic acid (PA), and butyric acid (BA)] were measured using high performance liquid chromatography (HPLC) [column, Shodex RSpak KC-811S-DVB gel C (8.0 mm × 30 cm; Shimadzu, Tokyo, Japan); oven temperature, 50°C; mobile phase, 3 mmol/L HClO4; flowrate, 1.0 mL/min; injection volume, 5 μL; and detector, SPD-M10AVP] according to the conditions and technique published by Zhang et al. (2021).

### Analysis of protein fractions and other chemical composition

The remaining silage samples were dried at 65°C for 48 h until a constant weight was achieved to measure the DM content. The determination of neutral detergent fiber (NDF) and acid detergent fiber (ADF) were based on the principle of the Van Soest fiber analysis (Van Soest et al., 1991). Colorimetry was used to determine the WSC content following a reaction with an anthrone reagent. Crude protein (CP) content was analyzed using the method described by the Association of Official Analytical Chemists. The TP and NPN contents were measured according to the method described by Licitra et al. (1996). The phenol-hypochlorite test was used to determine the NH_3_-N concentration.

### *In vitro* rumen fermentation

*In vitro* gas production at 72 h and *in vitro* dry matter digestibility at 48 h were performed by the Beijing Zhengfang Xingda Technology Development Co (Beijing, China). Silage samples (220 mg of DM) were weighed into 120 mL glass bottles in advance, with each treatment carried out using six replicates. 30 mL of incubation fluid was then added to each bottle and the gas volumes recorded at hours 0, 2, 4, 6, 8, 10, 12, 16, 20, 24, 30, 36, 42, 48, 54, 60, and 72 of incubation. The same samples were incubated at 39°C for 48 h. The fermentation slurry was filtered from each bottle using a pre-weighed nylon bag to collect the residue, dried at 65°C to a constant weight to calculate *in vitro* dry matter digestibility and *in vitro* crude protein digestibility.

### Statistical analysis

The statistical analyses were conducted using SPSS Statistics 21 software. The effects of the different treatments were evaluated using two-way ANOVA analysis of variance, with Duncan’s multiple range tests. A significance value of *P* < 0.05 was set.

## Results

### Screening results

A total of 264 strains were isolated and purified from silages of *N. cadamba* leaves ensiled for 29 days, grape pomace, banana leaves, mangosteen fruit pericarps, and *N. cadamba* leaves ensiled for 4 days. Supplementary Table 1 shows the pH value ranges of the strains isolated from the silages. Twenty-nine strains of high acid producing bacteria were selected from the five silages. Supplementary Table 2 shows the results of the 29 strains. After sequence alignment using the BLAST, all 29 strains screened were shown to be LP. The datasets presented in this study can be found in online repositories of the NCBI. Tannin tolerance tests were then carried out at different concentrations (0, 1, 2, and 4%) of TA. Supplementary Table 3 showed the results of these tannin tolerance tests. Finally, four LP strains with better tannin tolerance were selected and named LP1, LP2, LP3, and LP4. Of these, LP4 from mangosteen shells had the highest viable count in the 4% tannin tolerance test.

### Characteristics of the silage materials

The chemical content and microbial population of the stylo and WPS before ensiling are listed in Table 1. The DM of stylo was 32.91%. The basic nutritional indicators such as CP, NDF, and ADF were 8.93% DM, 68.60% DM, and 52.90% DM, respectively. The WSC content of WPS was 1.23% DM. The number of bacteria such as LAB, CB, yeasts, and molds were 5.48 log_10_ colony forming units per gram, 6.44 log_10_ cfu/g, 4.29 log_10_ cfu/g, and 3.40 log_10_ cfu/g, respectively. The DM of WPS was 28.27% DM. The basic nutritional indicators such as CP, NDF, and ADF were 24.07% DM, 40.64% DM, and 23.40% DM, respectively. The WSC content of soybean was 2.39% DM. The number of bacteria such as LAB, CB, yeasts, and molds were 6 log_10_ cfu/g, 5.98 log_10_ cfu/g, 5 log_10_ cfu/g, and 3.72 log_10_ cfu/g, respectively.

**TABLE 1:** The chemical compositions and microbial population of stylo and whole-plant soybean (WPS) before ensiling (Mean ± SD, *n* = 3).

Items	*Stylosanthes*	Whole-plant soybean
Dry matter (%)	32.9 ± 0.22	28.3 ± 0.97
Crude protein (% DM)	8.93 ± 0.37	24.1 ± 1.62
Neutral detergent fiber (% DM)	68.6 ± 1.61	40.6 ± 2.46
Acid detergent fiber (% DM)	52.9 ± 0.87	23.4 ± 0.68
Water-soluble carbohydrate (% DM)	1.23 ± 0.17	2.39 ± 0.43
Lactic acid bacteria (LAB, log_10_ cfu/g FM)	5.48 ± 0.47	6.00 ± 0.10
Coliform bacteria (log_10_ cfu/g FM)	6.44 ± 0.20	5.98 ± 0.83
Yeast (log_10_ cfu/g FM)	4.29 ± 0.08	5.00 ± 0.05
Mold (log_10_ cfu/g FM)	3.40 ± 0.35	3.72 ± 0.13

DM, dry matter; FM, fresh matter; cfu, colony forming unit.

### Fermentation parameters of stylo silage

The effects of addition of LP and TA on fermentation parameters and microbial quantity of silage of stylo are shown in Table 2. Compared to the control group, the silage pH was reduced to varying degrees by the addition of LP strains or by the addition of higher concentrations of TA (*P* < 0.05). The pH of most of the 1% TA treatment groups was slightly higher compared to that of the corresponding 2% TA treatment groups (*P* > 0.05). The LP4 + 2% TA and LP3 + 1% TA groups had low pH levels. All the treatment groups had an increase to some extent in the number of LAB, compared to that of the control group, especially the groups with a mixture of 1% TA and LP added. In terms of LA, there was no significant difference between the treatment groups (*P* > 0.05), although the treatment groups without TA addition had a relatively higher level. However, in terms of BA, this was lower in all treatment groups compared to that measured in the control group. In this trial, BA was decreased in all treatment groups and was significantly (*P* < 0.05) lower in all LP except LP1, both alone or with the addition of the TA combination to the LP treatment groups.

**TABLE 2:** Effects of the addition of lactic acid bacteria (LAB) and tannic acid on the fermentation parameters and microbial quantity of silage of *Stylosanthes*.

Items	pH	LAB	CB	Yeast	Molds	LA	AA	PA	BA
			
		log_10_ cfu/g FM				% DM			
CK	5.04^a^	7.83^e^	<3.00	<2.00	<2.00	0.22^ab^	0.51^a^	0.04^ab^	2.51^a^
1%TA	5.07^a^	8.22^abcd^	<3.00	<2.00	<2.00	0.19^ab^	0.35^ab^	0.06^a^	1.97^ab^
2%TA	4.90^b^	7.86^e^	4.05	2.30	<2.00	ND	0.17^bcd^	0.00^b^	1.60^abc^
LP1	4.89^b^	8.03^cde^	<3.00	<2.00	<2.00	0.27^ab^	0.37^ab^	0.03^ab^	1.67^abc^
LP2	4.79^cd^	8.11^bcde^	<3.00	<2.00	<2.00	0.30^a^	0.30^abcd^	0.01^b^	1.26^bcd^
LP3	4.79^c^	8.11^bcde^	<3.00	<2.00	<2.00	0.20^ab^	0.23^bcd^	0.01^b^	0.94^bcd^
LP4	4.87^b^	8.09^bcde^	<3.00	<2.00	<2.00	0.24^ab^	0.32^abc^	0.01^b^	1.32^bcd^
LP1 + 1%TA	4.76^cdef^	8.43^a^	<3.00	<2.00	<2.00	0.23^ab^	0.25^bcd^	0.04^ab^	1.06^bcd^
LP1 + 2%TA	4.75^def^	7.91^e^	<3.00	<2.00	<2.00	0.07^b^	0.04^d^	0.03^ab^	0.23^d^
LP2 + 1%TA	4.78^cde^	8.37^ab^	<3.00	<2.00	<2.00	0.07^b^	0.06^cd^	0.01^b^	0.29^d^
LP2 + 2%TA	4.77^cdef^	8.11^bcde^	<3.00	<2.00	<2.00	0.08^ab^	0.07^cd^	0.02^ab^	0.38^d^
LP3 + 1%TA	4.74^ef^	8.33^ab^	<3.00	<2.00	<2.00	0.16^ab^	0.17^bcd^	0.03^ab^	0.71^cd^
LP3 + 2%TA	4.75^def^	8.12^bcde^	<3.00	<2.00	<2.00	0.15^ab^	0.14^bcd^	0.04^ab^	0.76^cd^
LP4 + 1%TA	4.78^cdef^	8.31^abc^	<3.00	<2.00	<2.00	0.15^ab^	0.19^bcd^	0.03^ab^	0.77^cd^
LP4 + 2%TA	4.74^f^	8.01^de^	<3.00	<2.00	<2.00	0.14^ab^	0.13^bcd^	0.04^ab^	0.54^cd^
SEM	0.02	0.03	–	–	–	0.02	0.03	0.00	0.12
*P*-value	0.000	0.000	–	–	–	0.284	0.007	0.238	0.001

LAB, lactic acid bacteria; CB, Coliform bacteria; LA, lactic acid; AA, acetic acid; PA, propionic acid; BA, butyric acid; cfu, colony forming unit; DM, dry matter; FM, fresh matter; CK, the control; ND, not detected; –, default; SEM, standard error of means. Means with different superscripts in the same column (a–f) differ (*P* < 0.05).

### Fermentation parameters of whole-plant soybean silage

The effects of the addition of LP and TA on the fermentation parameters and microbial quantity of WPS silage are shown in Table 3. The pH levels of all the treatment groups were lower than that measured in the control group, with the TA-LP4 treatment groups having the lowest pH values. All treatment groups had significantly lower pH (*P* < 0.01) except for the 1% TA alone treatment group. However, the overall pH was higher, with a pH below 4.2 a value that was considered to be better. The LAB counts were better in the LP alone treatment groups, especially LP2 and LP3. The LAB counts were also higher in the LP treatment groups with the addition of 1% TA than in the groups with the addition of 2% TA in all the TA combinations (*P* < 0.01), although the differences in the TA combined with LP4 treatment groups were not significant. All treatment groups had higher CB counts except for the LP2 + 2% TA and LP4 + 2% TA groups. In short, the addition of TA combined with LP4 treatment groups, especially LP4 + 2% TA, were better than that measured in the other groups.

**TABLE 3:** Effects of the addition of lactic acid bacteria and tannic acid on the fermentation parameters and microbial quantity of silage of soybean.

Items	pH	LAB	CB	Yeasts	Molds	LA	AA	PA	BA
			
		log_10_ cfu/g FM				% DM			
CK	5.77^a^	8.66^cd^	6.31^a^	<2.00	<2.00	0.32^b^	0.68^b^	ND	2.44
1%TA	5.71^a^	8.46^de^	6.31^a^	<2.00	<2.00	0.37^b^	1.37^ab^	ND	5.81
2%TA	5.49^b^	8.35^ef^	6.14^a^	<2.00	<2.00	0.54^ab^	0.89^ab^	ND	3.72
LP1	5.19^de^	8.88^ab^	5.17^abc^	<2.00	<2.00	0.66^ab^	1.76^ab^	ND	4.03
LP2	5.39^bc^	9.02^a^	5.67^ab^	<2.00	<2.00	1.23^ab^	1.98^ab^	ND	4.21
LP3	5.27^cd^	8.95^a^	5.53^abc^	<2.00	<2.00	1.53^a^	2.15^ab^	ND	5.21
LP4	5.20^de^	8.86^ab^	4.72^bc^	<2.00	<2.00	0.69^ab^	1.01^ab^	ND	3.23
LP1 + 1%TA	4.99^fg^	8.56^cd^	4.30^c^	<2.00	<2.00	0.52^ab^	0.87^b^	ND	1.74
LP1 + 2%TA	5.20^de^	8.24^fg^	5.84^ab^	<2.00	<2.00	1.07^ab^	2.58^a^	ND	4.99
LP2 + 1%TA	5.14^def^	8.64^cd^	4.74^bc^	<2.00	<2.00	0.51^ab^	1.21^ab^	ND	2.08
LP2 + 2%TA	5.00^fg^	8.34^ef^	<3.00	<2.00	<2.00	0.32^b^	0.64^b^	ND	1.62
LP3 + 1%TA	5.09^ef^	8.70^bc^	4.79^bc^	<2.00	<2.00	0.74^ab^	1.16^ab^	ND	2.85
LP3 + 2%TA	5.15^def^	8.17^fg^	4.64^bc^	<2.00	<2.00	0.65^ab^	1.63^ab^	ND	3.38
LP4 + 1%TA	4.88^gh^	8.17^fg^	4.30^c^	<2.00	<2.00	0.90^ab^	1.43^ab^	ND	2.71
LP4 + 2%TA	4.75^h^	8.12^g^	<3.00	<2.00	<2.00	0.43^b^	0.56^b^	ND	1.34
SEM	0.04	0.05	0.14	–	–	0.08	0.14	–	3.78
*P*-value	0.000	0.000	0.003	–	–	0.248	0.189	–	0.638

LAB, lactic acid bacteria; CB, Coliform bacteria; LA, lactic acid; AA, acetic acid; PA, propionic acid; BA, butyric acid; cfu, colony forming unit; DM, dry matter; FM, fresh matter; CK, the control; ND, not detected; –, default; SEM, standard error of means. Means with different superscripts in the same column (a–g) differ (*P* < 0.05).

### Protein composition of the silages

The effects of addition of LP and TA on the protein contents of the silage of stylo and WPS are shown in Table 4. In terms of CP content, only LP4 increased the CP content of stylo. For WPS, all four LP strains increased the CP content, but reduced the proportion of TP and NPN in total N for WPS. However, for stylo, adding each of the four LP strains increased both TP and NPN contents. The addition of TA alone increased TP content and decreased NPN and NH_3_-N contents, although the addition of TA with LP groups, particularly the LP4 + 2% TA treatment, achieved better results.

**TABLE 4:** Effects of the addition of lactic acid bacteria and tannic acid on the protein contents of silage of stylo and soybean.

Items	CP (%DM)		TP (%TN)		NPN (%TN)		NH_3_-N (%TN)	
	Stylo	WPS	Stylo	WPS	Stylo	WPS	Stylo	WPS
CK	8.82^abc^	18.9^abcde^	52.7^g^	37.7^h^	47.3^a^	62.3^b^	8.91^a^	17.5^a^
1%TA	8.86^abc^	18.4^abcdef^	60.0^defg^	48.6^bcd^	40.0^abcd^	51.4^fgh^	6.64^bc^	10.6^b^
2%TA	8.29^cd^	17.7^bcdef^	64.4^cdef^	53.7^a^	35.6^bcde^	46.3^i^	4.54^d^	6.36^bcd^
LP1	8.65^abcd^	21.5^ab^	59.5^efg^	32.3^i^	40.5^abc^	67.7^a^	6.96^c^	10.5^b^
LP2	8.24^cd^	20.5^abc^	58.1^fg^	33.3^i^	41.9^ab^	66.8^a^	6.02^c^	12.3^b^
LP3	8.45^bcd^	22.5^a^	58.8^fg^	31.9^i^	41.2^ab^	68.1^a^	5.86^c^	9.50^bc^
LP4	9.50^a^	20.3^abcd^	56.0^g^	30.4^i^	44.0^a^	69.6^a^	6.98^c^	9.11^bcd^
LP1 + 1%TA	8.63^abcd^	18.1^bcdef^	66.9^bcde^	40.7^gh^	33.1^cdef^	59.3^bc^	3.42^e^	4.51^ef^
LP1 + 2%TA	9.37^ab^	15.4^ef^	65.5^cdef^	49.1^bc^	34.5^bcde^	50.9^gh^	0.88^g^	4.19^ef^
LP2 + 1%TA	8.66^abcd^	16.1^def^	65.3^cdef^	45.0 ^def^	34.7^bcde^	55.0^def^	3.66^e^	5.99^de^
LP2 + 2%TA	8.26^cd^	14.4^f^	73.1^ab^	50.6^ab^	26.9^fg^	49.4^hi^	1.98^f^	3.64^ef^
LP3 + 1%TA	8.51^bcd^	17.7^bcdef^	65.8^bcdef^	41.9^fg^	34.2^bcdef^	58.1^cd^	3.17^e^	5.52^e^
LP3 + 2%TA	7.78^d^	16.2^cdef^	76.2^a^	46.0^cde^	23.8^g^	54.0^efg^	2.00^f^	4.64^ef^
LP4 + 1%TA	8.81^abc^	20.4^abcd^	67.4^bcd^	44.3^efg^	32.7^def^	55.7^cde^	3.66^e^	2.88^ef^
LP4 + 2%TA	8.91^abc^	21.8^ab^	70.0^abc^	51.8^ab^	30.0^efg^	48.2^hi^	1.75^f^	1.89^f^
SEM	0.09	0.45	1.08	1.19	1.08	1.19	0.35	0.66
*P*-value	0.031	0.001	0.000	0.000	0.000	0.000	0.000	0.000

CP, crude protein; DM, dry matter; TP, true protein; %TN, presented as its accounting proportion in total nitrogen; NPN, non-protein nitrogen; NH_3_-N, ammonia-nitrogen; Stylo, *Stylosanthes* silage; WPS, the whole-plants soybean silage; CK, the control; SEM, standard error of means. Means with different superscripts in the same column (a–i) differ (*P* < 0.05).

### *In vitro* rumen fermentation characteristics of the silages

**FIGURE 1:**
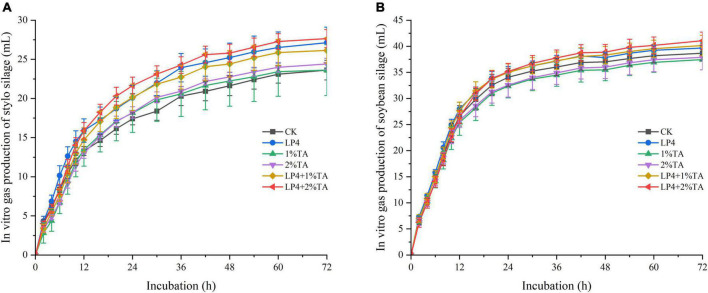
*In vitro* gas production and dry matter digestibility of different treatments of *Stylosanthes* silage **(A)** and whole-plant soybean (WPS) silage **(B)** in 72 h.

**FIGURE 2:**
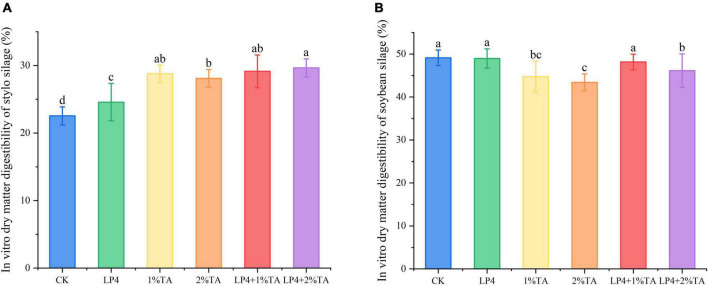
*In vitro* dry matter digestibility of *Stylosanthes* silage **(A)** and whole-plant soybean (WPS) silage **(B)** in 48 h. Different letters over the bars indicate significant differences (*P* < 0.05).

**FIGURE 3:**
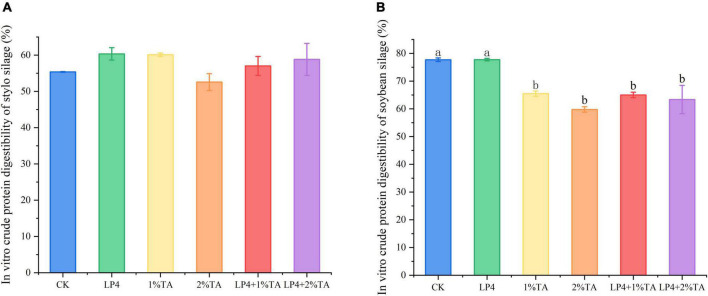
*In vitro* crude protein digestibility of *Stylosanthes* silage **(A)** and whole-plant soybean (WPS) silage **(B)** in 48 h. Different letters over the bars indicate significant differences (*P* < 0.05).

The addition of TA combination with LP strains was effective for increasing the total *in vitro* gas production compared to that measured in the control (Figure 1). Similarly, *in vitro* dry matter digestibility (Figure 2) and crude protein digestibility (Figure 3) of stylo were increased. The *in vitro* crude protein digestibility of the WPS treatments with combined addition of LP4 and TA were lower than in the control group.

## Discussion

### Characteristics of silage materials

Compared with other experiments, the DM and fiber contents of stylo in this experiment were higher, whereas the CP and WSC contents were at a lower level (Li et al., 2017; Wang C. et al., 2020; Pitiwittayakul et al., 2021). It was possible that the stylo was collected at blooming stage, resulting in less than excellent characteristics of the materials. Most of the value of chemical composition of fresh WPS in the present study were in the range for soybean reported by others (Zeng et al., 2020; Bolson et al., 2021; Silva et al., 2021; Gandra et al., 2022). In short, neither material had a high percentage of DM content, which was less than the ideal DM (40%) for good silage (Flythe and Russell, 2004). This may lead to marked effluent loss during ensiling (He et al., 2020d). Forages containing high levels of moisture also favor the fermentation of undesirable bacteria (He et al., 2020b). In addition, the WSC of both stylo and WPS were below the ideal WSC (5%) (Ni et al., 2017), with this potentially affecting the growth of LAB. However, the CP content of WPS in the present study was higher than that reported by previous studies. This might have been influenced by many factors, such as cultivar and harvest time. Compared with stylo, WPS had lower NDF and ADF content and higher WSC content, which facilitated the fermentation of the LAB. Moreover, the LAB counts of stylo and WPS met the standard for good silage feedstock as reported by Cai et al. (1998). However, the CB counts of stylo and WPS were also higher than 5 log_10_ cfu/g fresh matter, which may have affected silage quality. The appropriate addition of TA and LP might help to alleviate those problems.

### Fermentation parameters of stylo and whole-plant soybean silage

One of the indicators of silage fermentation quality is the pH value. The pH of high-quality silages should be between 3.8 and 4.2 (Kung and Shaver, 2001). The final pH of silage is influenced by a variety of parameters, the most important of which are the concentration of LA and the buffering capability of the plants (Kung et al., 2018). It is often difficult to achieve a pH value of 4.5 or lower in legume silage, while the final pH of soybean silage is generally around 4.5 (Nkosi et al., 2016). In the present study, the pH of both WPS and stylo was above 4.5. The high buffering capacity of stylo and WPS has been reported to slow down the decline in pH decline, lengthen the fermentation process, and increase the consumption of WSC (Kizilsimsek et al., 2017). However, in our study the WSC content of the material was too low to well complete fermentation, resulting in a high pH, which reduced the inhibition of undesirable bacteria, particularly *Clostridium*. In addition, a high DM content may also affect the pH value. Kung and Shaver (2001) reported that a DM content of less than 30% promoted Clostridial fermentation, which led to a pH higher than 4.8. The high concentration of BA in the treatment groups of WPS in our trial may also have resulted in this outcome (Kung and Shaver, 2001). BA is a fermentation indicator for *Clostridium perfringens*, with levels above 5 g/kg DM (0.5% DM) providing evidence of significant activity of *Clostridium* in silage that prompts livestock to reduce their intake of forage (Muck, 2010).

The experiments in this study showed that adding LP or 2% TA alone significantly reduced the pH of silage, although the combination of LP and TA was better (*P* < 0.01), particularly LP4 + 2% TA. The addition of TA enhanced the LA and AA content of WPS to the same extent as LP alone, a result consistent with previous studies (Wang et al., 2021). However, the addition of TA combined with LP produced different results, possibly due to the influence of epiphytes.

Common bacterial communities in silage include LAB, CB, yeasts, and molds. In general, LAB are the principal microorganisms that influence silage fermentation by creating organic acids that have a preservative effect. The LAB count must be greater than 5 log_10_ cfu/g FM for excellent silage preservation (Yang et al., 2016). CB is a strong competitor of LAB and is responsible for lower amino acid concentration in silages, resulting in nutritional loss (Rauramaa et al., 1987). The control groups of stylo and WPS had a higher BA content and in addition WPS silage had a high CB count. CB can cause illness in animals and is also responsible, in part, for NH_3_-N formed from protein degradation and NO_3_ reduction in silage (Muck, 2010). Therefore, the quantity of CB should be minimized. This study showed that the combined addition of LP and TA inhibited CB to a greater extent than the addition of 2% TA or LP alone, and that the best results were obtained with LP2 + 2% TA and LP4 + 2% TA.

Lactic acid, acetic acid, propionic acid, and butyric acid are the main organic acids in silages (Kung and Shaver, 2001). Excellent quality silage has high levels of LA and PA, small amounts of AA, and no or very small amounts of BA (Li et al., 2012). The concentrations of LA in legume silages were reported to be 20–40 g/kg DM (2–4% DM) (Soundharrajan et al., 2019). However, all treatment groups of stylo and WPS in our experiment did not reach 2%. This may have been attributable to the effect of the raw material, because the stylo and WPS in the other experiments did not meet that criterion (He et al., 2021; Wang et al., 2021). The large quantities of AA, PA, and BA in the total organic acid indicated substantial heterofermentation, implying that the fermentation process needs to be improved (He et al., 2020d). The addition of LP alone could reduce the ratio of AA, PA, and BA in total organic acid, while the combined addition of LP and TA, especially LP4 + 2% TA, could further increase the ratio of LA in the total organic acid to twice or more than that measured in the control groups. The ratio of LA to AA is an indicator of the extent of homofermentation in relation to heterofermentation during ensiling (Wang et al., 2019). With the combined addition, the LA content of stylo was decreased while AA content was decreased even more, ultimately leading to an increased ratio. The LA to AA values of WPS were also upgraded from 1:2 to around 4:5. In addition, BA, an indicator of the influence of silage smell and intake, was also decreased significantly (*P* < 0.01). Those results indicated that the additives were effective.

### Effects of additives on the protein composition of silage

Proteolysis is a common issue in ensiling high-protein forages like stylo and soybean. During silage, proteins are hydrolyzed by plant proteases into peptides and free amino acids, which are then further degraded to amides, amines, and ammonia by microbial activity (Kung et al., 2018). Therefore, NPN and NH_3_-N are the important indicators of hydrolysis. In addition, the NH_3_-N content indicates the activity of undesirable microorganisms like *Clostridium*. NPN has lower nitrogen use efficiency than TP, and therefore a high content of NPN in animal feed is undesirable (He et al., 2020c). Factors currently known to affect proteolysis are the species in the forage, the stage of maturity of the crop, the wilting level, and the rate of decline in silage pH (Tabacco et al., 2006). Epiphytic microorganisms or additives may also have an effect on hydrolysis.

The high proportion of NPN in the total nitrogen (47.3 and 62.3%) in the control groups of stylo and WPS in the current study indicates severe proteolysis in the stylo and WPS silages. The higher buffering capacity of the legumes slowed down the decrease in pH, which weakened the inhibitory effect on undesirable bacteria. In addition, the low DM content (<30% DM) and high moisture content of the raw material also promoted the growth and fermentation of *Clostridium* (Kung and Shaver, 2001). Under these two conditions, excessive protein decomposition occurs, producing higher levels of alkaline substances such as ammonia and nitrogen, leading to higher ammonia concentrations (12–15% of CP) and pH values, which explains the high pH of WPS silage (Wang et al., 2021).

There is evidence that TA can reduce proteolysis (He et al., 2020b; Wang et al., 2021). Inoculation with LP can also reduce the proportion of NH_3_-N leading to reduced proteolysis (Li et al., 2017). LP can facilitate a rapid reduction in pH for the purpose of inhibiting spoilage bacteria. The addition of LP alone had different effects on the two silages in terms of CP, TP, and NPN content. This variation might be useful for stylo silage and detrimental to WPS silage, because the inoculated LP competitively inhibited epiphytic LAB of WPS. This effectively inhibited the growth of CB, especially in the LP4 + 2% TA treatment group of WPS, as the experiment included inoculation of tannin tolerance LP, which could tolerate certain concentrations of TA. In the present study, the NH_3_-N and NPN contents were decreased according to the dose of TA in combination with LP rather than by TA and LP alone (*P* < 0.01).

### Effect on *in vitro* gas production, dry matter digestibility, and crude protein digestibility

*In vitro* gas production, dry matter digestibility, and crude protein digestibility of the silages are shown in Figures 1–3. The rumen of a ruminant is a complex anaerobic fermentation system. During fermentation, the feed is degraded by microorganisms in the rumen, producing gases such as methane, carbon dioxide, and hydrogen (Getachew et al., 2004). Typically, the cumulative gas production is an indicator of the microbial activity (Ai et al., 2013), with production related to the level of nutrients in the rumen and the composition of the diet. The higher the content of fermentable nutrients in the diet, the higher the microbial activity and total production of fermented gas (Qiao et al., 2015; Sheng et al., 2019). This conclusion is consistent with the results of our experiment. The NDF of WPS was lower than the NDF of stylo, while the 24-h gas production of WPS was higher than that of stylo. This is in agreement with the experiments of Ai et al. (2013) who showed that lower fiber content, higher CP, and nitrogen-free extract (NFE) content can lead to higher *in vitro* fermentation. Feeds with a CP content of less than 10% lead to reduced microbial activity in the rumen, resulting in reduced gas production (Kilic and Garipoglu, 2009). This may account for the low gas production we observed in the stylo treatment groups. The addition of TA has been shown to reduce *in vitro* gas production of silage (Chen et al., 2022), which is consistent with the performance of the WPS-treated groups in our experiment. This might be because TA inhibits microbial activity in the rumen and reduces digestion and utilization of fermentation substrates, thereby reducing rumen gas production (Wang J. et al., 2020). However, the stylo treatment groups in our study showed different results, which might have been influenced by the epiphytic bacteria of stylo. The combined addition of LP4 and TA increased *in vitro* gas production of both stylo and WPS silage.

One of the most important factors determining forage intake is digestibility, which is determined mainly by the chemical composition (He et al., 2018). The *in vitro* dry matter digestibility of the stylo and WPS silages for 48 h are shown in Figure 2. *In vitro* dry matter digestibility is an important factor that influences the dry matter intake of ruminants, with higher DM digestibility indicating that the material is more easily digested and utilized by ruminants (Shengchen et al., 2017). The DM content and fiber content can affect *in vitro* dry matter digestibility, with the rumen of ruminants digesting fresh, juicy forage more than fibrous forage (Yao et al., 2021). We observed that there was a significant increase (*P* < 0.01) in the digestibility levels of stylo when LP and TA were added to the silage, although the digestibility of WPS was decreased. This might be because the CP content of WPS was higher, which can influence *in vitro* digestibility (Cherdthong and Wanapat, 2013).

Increasing the amount of available crude protein in the rumen (uCP) is an important basis for improving animal production performance. In the Cornell Net Carbohydrate and Protein System (CN), TP component can be divided depending on the rate of rumen degradation into PB_1_ (fast degradation), PB_2_ (medium degradation), and PB_3_ (slow degradation) (Guo et al., 2013). A portion of PB_2_ and most of PB_3_ pass through the rumen into the back part of the digestive tract. In addition, the tannin–protein complex, also known as PC (binding protein), reaches the abomasum and small intestine and releases protein because of a change in pH value. With the addition of LP4 and 2% TA, *in vitro* crude protein digestibility of WPS silage was reduced significantly by 18% (*P* < 0.01), while the increase in *in vitro* crude protein digestibility of stylo silage was also reduced. Taken collectively, the addition of 2% TA combined with LP was more effective.

## Conclusion

The present study illustrated that ensiling stylo and WPS with the addition of TA together with LP, especially LP4 + 2% TA, decreased the number of undesirable microorganisms and contents of BA, non-protein nitrogen, and ammonia-nitrogen and effectively increased the TP content. This indicates that the addition of TA together with LP can effectively inhibit undesirable fermentation and protein degradation of high protein silage, enhance the quality of silage fermentation, and protein reservation rates. Furthermore, the addition of TA with LP was also effective for increasing the *in vitro* gas production of stylo and WPS silages and decreasing *in vitro* crude protein digestibility of WPS silage. The effects of this addition on digestion and absorption of the animals should be studied in greater detail in future research.

## Data availability statement

The sequencing data were submitted to the NCBI Sequence Read Archive database (accession numbers can be found in Supplementary Table S2).

## Author contributions

XG designed the experiments. LFG provided the silage materials. SW collected the samples. XG, LG, DC, and SW analyzed the data. LG wrote the manuscript. WZ, RP, and QZ helped supervise the manuscript writing. All authors read and approved the final manuscript.
