# Supplementary material for: Tannin tolerance lactic acid bacteria screening and their effects on fermentation quality of stylo and soybean silages
Source: Front Microbiol. 2022 Sep 15;13:991387. doi: 10.3389/fmicb.2022.991387 (PMC9520722; doi:10.3389/fmicb.2022.991387)
Supplement: Supplementary file 1 [file Data_Sheet_1.docx]

**Table S1** Comparison of pH value ranges of lactic acid bacteria screened from different silage materials

| Range of pH | NCL1 | | Grape pomace | | Banana leaves | | NCL2 | | Mangosteen hulls | |
| --- | --- | --- | --- | --- | --- | --- | --- | --- | --- | --- |
|  | Strains counts | raito | Strains counts | raito | Strains counts | raito | Strains counts | raito | Strains counts | raito |
| 3.31~3.40 | 4 | 4.00% | 0 | 0 | 0 | 0 | 0 | 0 | 0 | 0 |
| 3.41~3.50 | 66 | 66.0% | 0 | 0 | 0 | 0 | 0 | 0 | 0 | 0 |
| 3.51~3.60 | 30 | 30.0% | 0 | 0 | 1 | 2.00% | 27 | 79.5% | 24 | 60.0% |
| 3.61~3.70 | 0 | 0 | 4 | 10.0% | 10 | 20.0% | 1 | 2.90% | 4 | 10.0% |
| 3.71~3.90 | 0 | 0 | 17 | 42.5% | 10 | 20.0% | 2 | 5.90% | 6 | 15.0% |
| 3.91~4.10 | 0 | 0 | 1 | 2.50% | 19 | 38.0% | 3 | 8.80% | 2 | 5.00% |
| 4.11~4.30 | 0 | 0 | 0 | 0 | 9 | 18.0% | 0 | 0 | 2 | 5.00% |
| 4.31~4.50 | 0 | 0 | 2 | 5.00% | 0 | 0 | 0 | 0 | 0 | 0 |
| 4.51~4.70 | 0 | 0 | 2 | 5.00% | 0 | 0 | 1 | 2.90% | 0 | 0 |
| 4.71~4.90 | 0 | 0 | 5 | 12.5% | 0 | 0 | 0 | 0 | 0 | 0 |
| 4.91~5.10 | 0 | 0 | 6 | 15.0% | 1 | 2.00% | 0 | 0 | 2 | 5.00% |
| 5.11~5.30 | 0 | 0 | 3 | 7.50% | 0 | 0 | 0 | 0 | 0 | 0 |

NCL1: *Neolamarckia cadamba* leaves ensiling for 29 days; NCL2: *Neolamarckia cadamba* leaves ensiling for 4 days.

**Table S2** Identification results of screened lactic acid bacteria with high acid production

| Strain number | Sequence alignment | NCBI accession number |
| --- | --- | --- |
| 1 | *Lactobacillus plantarum* | ON254142 |
| 2 | *Lactobacillus plantarum* | ON254143 |
| 3 | *Lactobacillus plantarum* | ON254144 |
| 4 | *Lactobacillus plantarum* | ON254145 |
| 5 | *Lactobacillus plantarum* | ON254146 |
| 6 | *Lactobacillus plantarum* | ON254147 |
| 7 | *Lactobacillus plantarum* | ON254148 |
| 8 | *Lactobacillus plantarum* | ON254149 |
| 9 | *Lactobacillus plantarum* | ON254150 |
| 10 | *Lactobacillus plantarum* | ON254151 |
| 11 | *Lactobacillus plantarum* | ON254152 |
| 12 | *Lactobacillus plantarum* | ON254153 |
| 13 | *Lactobacillus plantarum* | ON254154 |
| 14 | *Lactobacillus plantarum* | ON254155 |
| 15 | *Lactobacillus plantarum* | ON254156 |
| 16 | *Lactobacillus plantarum* | ON254157 |
| 17 | *Lactobacillus plantarum* | ON254158 |
| 18 | *Lactobacillus plantarum* | ON254159 |
| 19 | *Lactobacillus plantarum* | ON254160 |
| 20 | *Lactobacillus plantarum* | ON254161 |
| 21 | *Lactobacillus plantarum* | ON254162 |
| 22 | *Lactobacillus plantarum* | ON254163 |
| 23 | *Lactobacillus plantarum* | ON254164 |
| 24 | *Lactobacillus plantarum* | ON254165 |
| 25 | *Lactobacillus plantarum* | ON254166 |
| 26 | *Lactobacillus plantarum* | ON254167 |
| 27 | *Lactobacillus plantarum* | ON254168 |
| 28 | *Lactobacillus plantarum* | ON254169 |
| 29 | *Lactobacillus plantarum* | ON254170 |

**Table S3** Live counts of lactic acid bacteria in tannin solution from five kinds of silages screening

| Strain number | pH | Tannic acid concentration (%) | | | |
| --- | --- | --- | --- | --- | --- |
|  |  | 0 | 1 | 2 | 4 |
| 1 | 3.42 | 7.18^abcde^ | 3.16^nop^ | 3.10^defg^ | 2.91^hijk^ |
| 2 | 3.41 | 7.3^ab^ | 4.92^bc^ | 3.36^bcdef^ | 3.20^efghi^ |
| 3 | 3.40 | 7.09^abcdef^ | 3^p^ | 2.38^hij^ | 3.02^ghijk^ |
| 4 | 3.42 | 7.38^ab^ | 3.1^op^ | 3.08^defg^ | 3.09^ghi^ |
| 5 | 3.42 | 7.43^a^ | 3.42^mnop^ | 2.33^hij^ | 3.19^efghi^ |
| 6 | 3.38 | 7.09^abcdef^ | 4.22^fghij^ | 2.00^j^ | 3.28^defgh^ |
| 7 | 3.42 | 7.19^abcde^ | 4.97^bc^ | 2.00^j^ | 3.50^cdef^ |
| 8 | 3.41 | 7.09^abcdef^ | 4.08^hij^ | 2.98^defgh^ | 3.55^cde^ |
| 9 | 3.38 | 6.56^hijk^ | 5.31^ab^ | 2.67^fghi^ | 3.38^defg^ |
| 10 | 3.37 | 7.36^ab^ | 4.66^cdef^ | 2.69^fghi^ | 3.05^ghij^ |
| **11** | **3.64** | **7.09^abcdef^** | **3.1^op^** | **3.94^ab^** | **4.20^a^** |
| 12 | 3.69 | 7.23^abcd^ | 3.52^lmno^ | 3.65^abcd^ | 3.26^defgh^ |
| **13** | **3.69** | **7.21^abcd^** | **3.46^lmno^** | **3.00^defgh^** | **3.79^bc^** |
| 14 | 3.62 | 6.82^cdefghi^ | 4.85^cd^ | 3.90^abc^ | 3.16^fghi^ |
| 15 | 3.73 | 7.07^abcdef^ | 4.82^cde^ | 2.80^efgh^ | 3.38^defg^ |
| 16 | 3.64 | 6.51^ijkl^ | 4.39^defghi^ | 2.85^efgh^ | 3.23^defgh^ |
| 17 | 3.64 | 7.03^abcdefg^ | 4.55^cdefg^ | 3.18^defg^ | 3.60^cd^ |
| 18 | 3.60 | 7.00^abcdefgh^ | 4.51^cdefgh^ | 2.98^defgh^ | 2.70^jk^ |
| 19 | 3.61 | 6.94^bcdefghi^ | 4.02^ijk^ | 2.58^ghij^ | 2.93^hijk^ |
| 20 | 3.61 | 7.25^abc^ | 4.14^ghij^ | 2.10^ij^ | 2.66^k^ |
| 21 | 3.54 | 6.59^ghijk^ | 3.98^ijk^ | 2.81^efgh^ | 2.82^ijk^ |
| 22 | 3.55 | 6.66^fghijk^ | 3.89^jkl^ | 3.25^cdefg^ | 3.23^defgh^ |
| 23 | 3.54 | 6.30^kl^ | 3.88^jkl^ | 3.43^bcde^ | 2.82^ijk^ |
| **24** | **3.54** | **6.75^efghij^** | **4.37^efghi^** | **4.13^a^** | **4.05^ab^** |
| 25 | 3.55 | 6.61^ghijk^ | 3.76^jklm^ | 3.14^defg^ | 2.99^ghijk^ |
| 26 | 3.58 | 7.08^abcdef^ | 3.58^klmn^ | 3.37^bcdef^ | 2.93^hijk^ |
| 27 | 3.58 | 6.78^defghi^ | 5.64^a^ | 2.97^defgh^ | 3.24^defgh^ |
| 28 | 3.58 | 6.32^jkl^ | 4.83^cde^ | 3.56^abcd^ | 3.18^efghi^ |
| **29** | **3.58** | **6.13^l^** | **4.54^cdefgh^** | **3.35^bcdef^** | **4.01^ab^** |

^1^Live counts of lactic acid bacteria (log_10_ colony forming units/mL).

^2^Means with different superscripts in the same column (a-p) differ (*P* < 0.05).
